# Performance evaluation of Cu_2_SrSnS_4_ based solar cell: effect of transition metal dichalcogenides buffer layer

**DOI:** 10.1038/s41598-025-91145-2

**Published:** 2025-02-25

**Authors:** Hanane Mebrek, Beddiaf Zaidi, Nourelhouda Mekhaznia, Hmoud Al-Dmour, Ali Barkhordari

**Affiliations:** 1 Laboratory of Radiation Physics, Department of Physics, Faculty of Material Sciences, University of Batna 1, Batna, Algeria; 2 Department of Physics, Faculty of Material Sciences, University of Batna 1, Batna, Algeria; 3 Department of Material Sciences, EchahidCheikhLarbiTebessi University, Tebessa, Algeria; 4 Department of Physics, Faculty of Science, Mutah University, Mutah, 61710 Jordan; 5 Faculty of Physics, Shahid Bahonar University of Kerman, Kerman, Iran

**Keywords:** Quaternary chalcogenide, Efficiency, MoS_2_, WS_2_, SCAPS 1-D, Optical physics, Techniques and instrumentation, Electronic properties and materials

## Abstract

The urgent demand for efficient renewable energy technologies has driven extensive research into quaternary chalcogenide materials, owing to their outstanding photovoltaic properties and potential for high performance. This study focuses on the design, performance optimization, and comparative analysis of Cu_2_SrSnS_4_-based solar cells, with particular emphasis on employing different transition metal dichalcogenide (TMD) buffer layers, specifically MoS_2_ and WS_2_. By utilizing SCAPS 1-D simulation software, the research systematically examines the impact of critical parameters such as buffer layer thickness, doping concentrations, and operating temperatures on the solar cell’s efficiency and stability. The simulation results demonstrate that the ZnO/MoS_2_/Cu_2_SrSnS_4_ configuration attained the highest efficiency, reaching an impressive 35.6%, significantly surpassing its counterpart with WS_2_ as the buffer layer, which achieved an efficiency of 29.1%. The findings demonstrate the significance of buffer layer selection and parameter optimization in maximizing the potential of Cu_2_SrSnS_4_ solar cells. Ultimately, this research offers valuable insights into the development of high-efficiency, stable photovoltaic technologies, advancing the future of next-generation quaternary chalcogenide solar cells.

## Introduction

The Cu_2_ZnSnS_4_ quaternary chalcogenide material has demonstrated excellent potential for photovoltaic applications due to its earth-abundant, non-toxic composition. However, its progress has been hindered by band-tailing effects, which destabilize the conduction and valence bands, limiting the efficiency of solar cells^1,2^. Specifically, antisite defects such as CuSn, SnCu, ZnSn, and SnZn introduce deep-level defect states within the bandgap, leading to potential fluctuations in the electronic band structure^3^. These defects cause band tailing, increase charge carrier recombination rates, and significantly limit fabricated devices’ open-circuit voltage (V_OC_)^4–6^. Although CZTS has been widely studied for photovoltaic applications^7^, its limitations necessitate exploring alternative quaternary materials with superior properties. Various studies have demonstrated the potential of CZTS-based solar cells through innovative structural and material combinations. For example, a CZTS-based device with ZnTe as a buffer layer achieved an impressive power conversion efficiency (PCE) of 23.47% in the Mo/CZTS/ZnTe/ZnO/ZnO: Al structure^8^. Similarly, integrating CZTS with a MASnI_3_ perovskite absorber (FTO/TiO_2_/MASnI_3_/CZTS/Au) yielded a J_SC_ of 31.66 mA/cm², V_OC_ of 0.96 V, and an efficiency of 20.28%^9^. However, band-tailing effects and antisite defects in CZTS continue to limit V_OC_ and overall efficiency, as seen in multi-absorber configurations such as CZTS/InSe, which only reached 16.30% efficiency^10^. These limitations motivate the search for alternative materials like CSTS, which offer improved defect tolerance and reduced band-tailing. To address these challenges, this study investigates Cu_2_SrSnS_4_ (CSTS), a novel alternative where Sr substitutes Zn. The larger ionic radius of Sr reduces band-tailing, which offers more stability and electronic properties. Theoretical calculations indicate that Cu_2_SrSnS_4_ (CSTS) exhibits a direct bandgap of approximately 1.79 eV^11,12^, high light absorption efficiency (with absorption coefficients over 10⁴ cm^− 1^)^13^, the abundance of non-toxic elements in its composition, and excellent defect tolerance, making it promising alternative for photovoltaic application.

According to previous reports, CSTS may also be a promising candidate for thin-film solar cells and PEC (photoelectrochemical) water-splitting applications, owing to its advantageous defect properties and superior optical characteristics^14,15^.

To further explore CSTS potential, we evaluated its performance in heterojunction solar cells, incorporating MoS_2_ and WS_2_ as buffer layers, compared to CZTS, which has been integrated with various buffer materials like CdS^16^, InSe^17^, and ZnTe^18^, CSTS paired with MoS_2_ or WS_2_ demonstrates a significant efficiency advantage. Showcasing its potential as a high-performance alternative considering realistic defect densities, recombination rates, and electron affinity. These transition metal dichalcogenides are known for their superior charge carrier transport properties, which can significantly enhance device efficiency^19,20^. Devices with two varied buffer layers, MoS_2_ and WS_2_, were analyzed. Under optimized conditions, the ZnO/MoS_2_/Cu_2_SrSnS_4_ and ZnO/WS_2_/Cu_2_SrSnS_4_ devices achieved photo conversion efficiencies exceeding 35.6% and 29.1%, respectively. Both devices exhibited comparable short-circuit current densities (J_SC_), though the MoS_2_ buffer layer delivered a higher open-circuit voltage (V_OC_). Overall, this study highlights Cu_2_SrSnS_4_ (CSTS) as a promising absorber material for high-efficiency heterojunction solar cells.

The inclusion of these transition metal dichalcogenides is significant, as they can enhance charge carrier transport and improve overall device efficiency. By comparing the results from both architectures, insights can be gained into the optimal design strategies for enhancing the efficiency of Cu_2_SrSnS_4_-based solar cells, potentially paving the way for their practical application in renewable energy technologies. This study explores the potential of CSTS as a high-performance absorber material for thin-film solar cells, addressing the challenges associated with CZTS. To achieve this, we analyze the material properties, evaluate the simulation setup using a solar cell capacitance simulator (SCAPS-1D)^21,22^, and assess the photovoltaic performance of devices using MoS_2_ and WS_2_ buffer layers. The findings are discussed to highlight their implications for optimizing CSTS-based solar cells, culminating in key conclusions and suggestions for future research directions.

## Device simulation

The two device architectures, ZnO/MoS_2_/Cu_2_SrSnS_4_ and ZnO/WS_2_/Cu_2_SrSnS_4_ were examined using SCAPS-1D software, with all simulations based on the standard AM 1.5G solar spectrum luminosity. Numerical simulations are essential for optimizing solar cell design, allowing researchers to predict the behavior of solar cells under various conditions before physical fabrication. In this study, the focus was on assessing the photovoltaic performance of Cu_2_SrSnS_4_-based solar cells, where MoS_2_ or WS_2_ were investigated as potential buffer layers.

Key variables such as absorber layer thickness, acceptor density, donor density, and operating temperature were systematically adjusted to determine their impact on the Cu_2_SrSnS_4_-based solar cells performance. All simulations were conducted at a temperature of 300 K under a 1.5 G solar spectrum, representing standard operating conditions, with a series resistance of 5 Ω and a shunt resistance of 300 Ω.

**Fig. 1:**
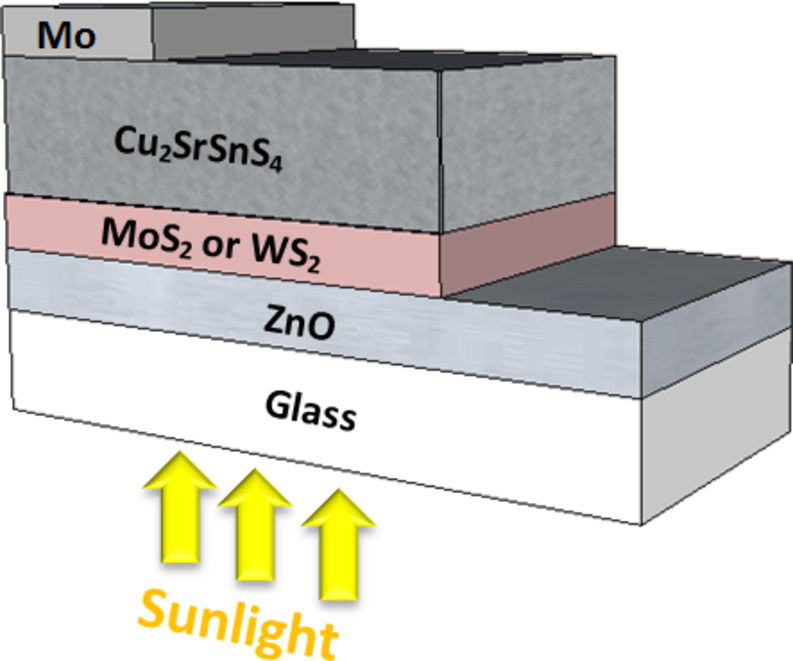
Configuration of ZnO/(MoS_2_ or WS_2_)/Cu_2_SrSnS_4_ solar cell used in the simulations.

**Fig. 2:**
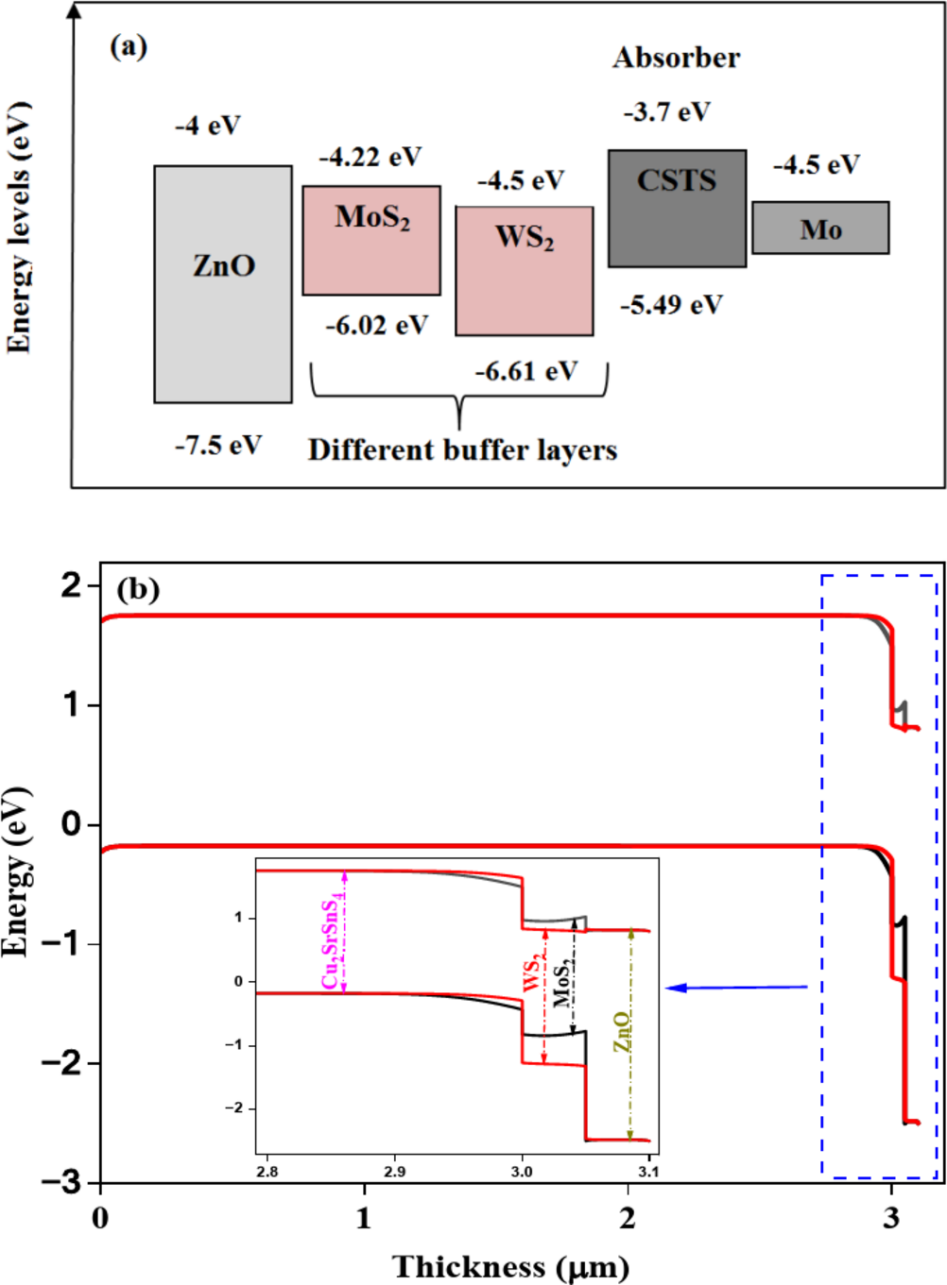
(**a**) Energy level alignment and (**b**) band diagrams of CSTS solar cells with MoS_2_ and WS_2_ buffer layer configurations.

The device structure is illustrated in Fig. 1, which consists of ZnO as the front reflector layer to enhance light transmission and reflection toward the absorber layer, followed by MoS_2_ or WS_2_ as buffer layers to reduce recombination losses and improve charge carriers transport, Cu_2_SrSnS_4_ as the absorber layer, and Mo is employed as the back contact to enhance charge extraction and the overall performance of the device. Figure 2 shows the energy band alignment and energy band diagram of the CSTS-based solar cell using various buffer layers.

**Fig. 3:**
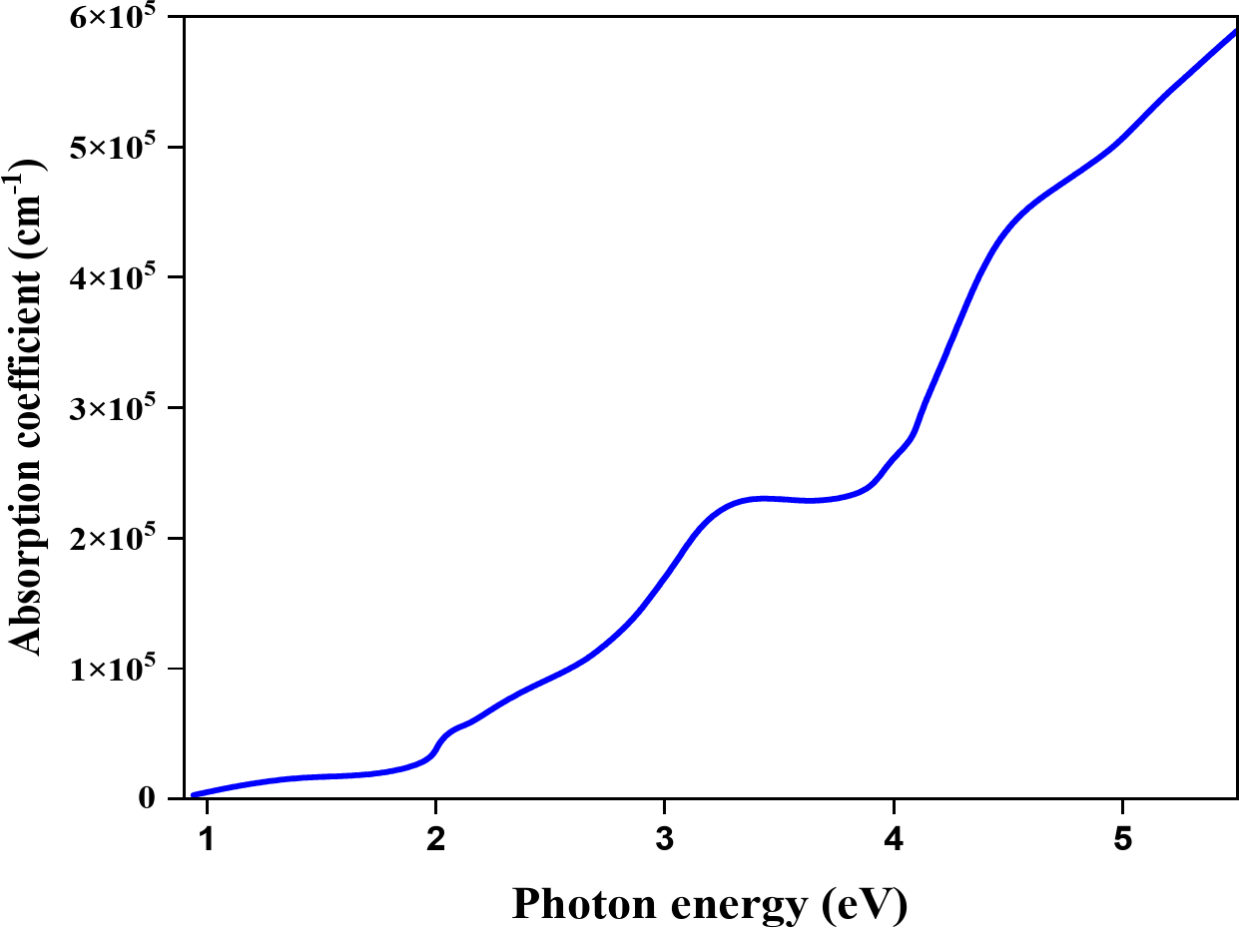
Absorption coefficient of CSTS as a function of photon energy, illustrating its optical properties and light absorption capability across various energy ranges^23^.

Figure 3 shows the absorption coefficient of CZTS observed (> 10^4^ cm^− 1^), confirming its excellent absorption properties as an absorber material for high-performance solar cells.

Table 1 provides the specifications for the device layers used in the ZnO/(MoS_2_ or WS_2_)/Cu_2_SrSnS_4_ simulations.

**Table 1:** Material parameters used in the numerical simulation.

Parameters	Cu_2_SrSnS_4_^24,25^	MoS_2_^26,27^	WS_2_^28^	ZnO^29^
Thickness (µm)	1 to 3	0.01 to 0.05	0.01 to 0.05	0.01
Gap energy (eV)	1.79	1.8	2.11	3.5
Electron affinity (eV)	3.7	4.22	4.5	4
Relative permittivity (eV)	5.92	13.5	13.5	9
$\:{\text{C}}_{\text{B}}$ density of states (cm^− 3^)	2.2 × 10^18^	2.2 × 10^17^	2.2 × 10^17^	2.2 × 10^18^
$\:{\text{V}}_{\text{B}\:}$density of states (cm^− 3^)	1.8 × 10^19^	1.8 × 10^19^	1.8 × 10^19^	2.2 × 10^18^
Mobility of electron (cm^2^/V.s)	100	100	100	100
Mobility of hole (cm^2^/V.s)	35	25	25	25

## Results and discussion

**Fig. 4:**
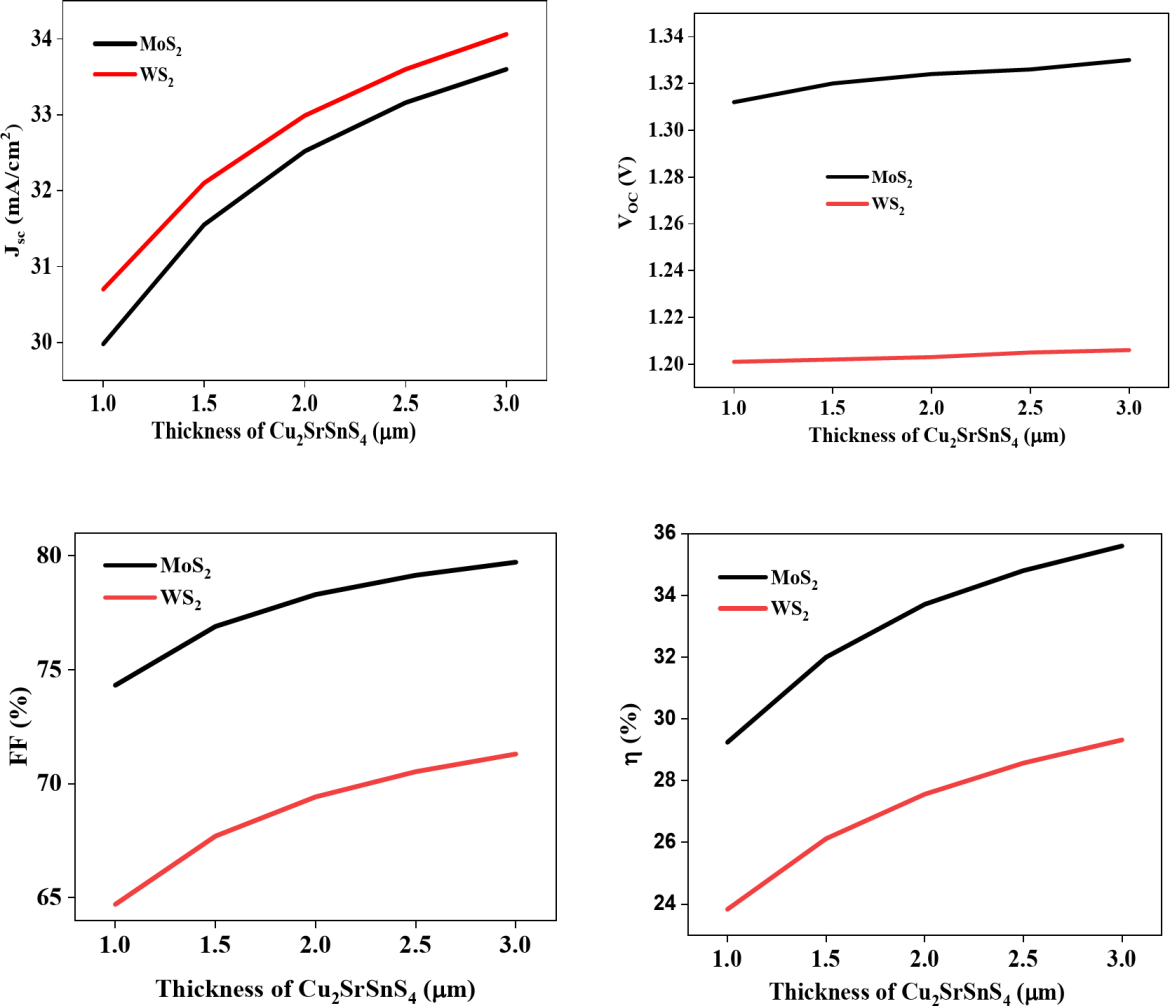
Effect of Cu_2_SrSnS_4_ thickness on the photovoltaic parameters.

Figure 4 illustrates the photovoltaic characteristics of the simulated structure as a function of the Cu_2_SrSnS_4_ layer thickness, ranging from 1 to 3 μm, and for different buffer layers. The results reveal that both the short-circuit current (J_SC_) and open-circuit voltage (V_OC_) increase rapidly before stabilizing. In thicker Cu_2_SrSnS_4_ thin films, the active layer captures more photons, thereby generating additional electron-hole pairs, as noted in prior studies^30^. This observation highlights the importance of absorber layer thickness in improving photon absorption^31^. Figure 4 indicates that the ideal Cu_2_SrSnS_4_ absorber thickness aligns with a significant rise in short-circuit current, identified at an optimum thickness of 3 μm. Increasing the active layer thickness from 1 to 3 μm results in a noticeable rise in short-circuit current density, from 29.98 to 33.6 mA/cm^2^ and 30.67 to 34.1 mA/cm^2^ for MoS_2_ and WS_2_ buffer layers, respectively. Meanwhile, the open-circuit voltage shows a linear improvement from 1.2 to 1.21 V and 1.3 to 1.34 V for WS_2_ and MoS_2_. It is observed that, at the optimal thickness, the open-circuit voltage is nearly identical between the two buffers. This indicates that short-circuit current primarily influences the performance of these solar cells. The simultaneous increase in J_SC_ and V_OC_ enhances both efficiency (η) and fill factor (FF). For the optimum thickness, efficiency (and fill factor) reaches 29.1% and 35.6% (71.19% and 79.62%) for WS_2_ and MoS_2_ buffers, respectively. The optimized performance parameters (V_OC_, J_SC_, FF, and η) for ZnO/MoS_2_/Cu_2_SrSnS_4_ and ZnO/WS_2_/Cu_2_SrSnS_4_ solar cells are based on values reported in the literature^32,33^.

**Fig. 5:**
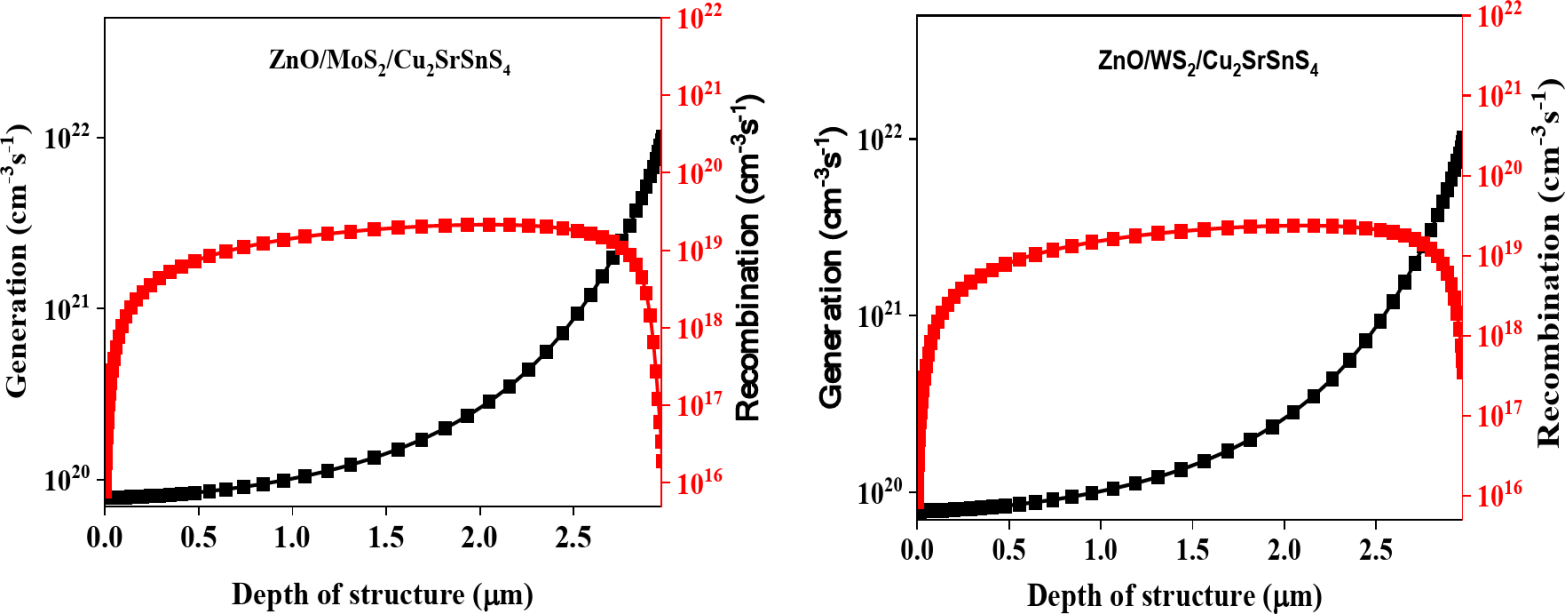
G-R profile of ZnO/(MoS_2_ or WS_2_)/Cu_2_SrSnS_4_ structure.

The generation-recombination (G-R) profile for ZnO/MoS_2_/Cu_2_SrSnS_4_ and ZnO/WS_2_/Cu_2_SrSnS_4_ solar cells, as simulated using SCAPS-1D (Fig. 5), reveals critical insights into charge generation and recombination mechanisms within these multi-layer structures. The SCAPS-1D simulations indicate substantial charge generation at the ZnO/MoS_2_/Cu_2_SrSnS_4_ and ZnO/WS_2_/Cu_2_SrSnS_4_ interfaces, which can be attributed to the high absorption coefficients of MoS_2_ and WS_2_ in the visible spectrum^34^. However, the G-R profile also highlights recombination losses at these interfaces, likely due to lattice mismatches or interface defects at the ZnO/MoS_2_ and ZnO/WS_2_ junctions. Such recombination hinders charge carrier transport and reduces both V_OC_ and FF.

Bulk recombination within the Cu_2_SrSnS_4_ absorber layer further degrades performance, as intrinsic defects act as trap states, reducing carrier lifetimes and contributing to efficiency loss^35^. Comparatively, the G-R profile suggests that the ZnO/WS_2_/Cu_2_SrSnS_4_ configuration may experience slightly lower recombination rates, potentially due to better energy alignment at the ZnO/WS_2_ interface, which could enhance charge extraction and transport. Addressing these recombination pathways through passivation-optimized buffer layers can improve photovoltaic efficiency in the designed solar cell. The significance of SCAPS-1D modeling in identifying these mechanisms emphasizes its role in improving material interfaces for better performance^36^.

**Fig. 6:**
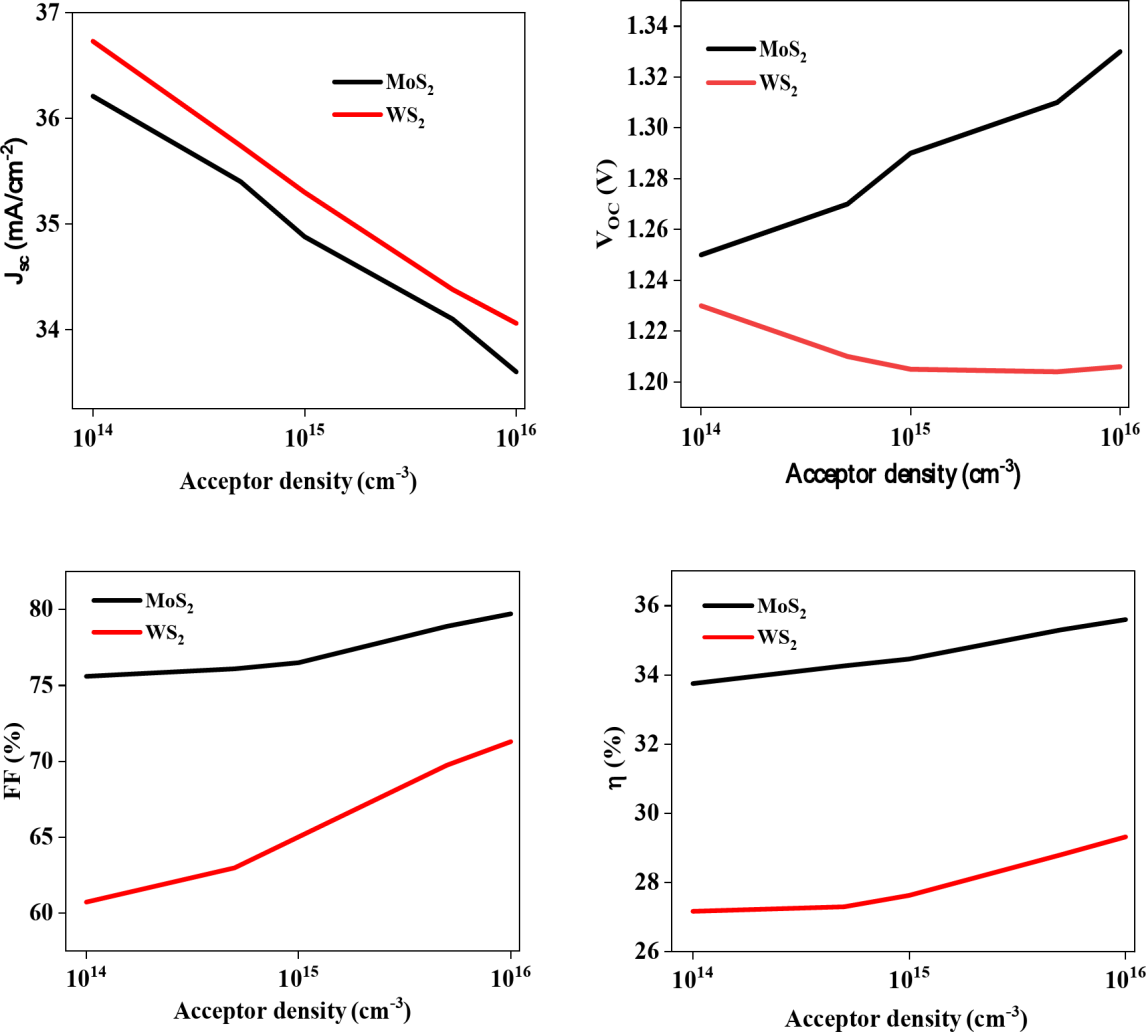
Cu_2_SrSnS_4_ acceptor density impaction the photovoltaic parameters.

Figure 6 illustrates the effect of varying the doping levels of the Cu_2_SrSnS_4_ absorber layer on key photovoltaic parameters. The absorber layer acceptor density values range from 10^14^ to 10^16^ cm^− 3^. Notably, an increase in the doping concentration correlates with a decline in these parameters. This trend indicates that higher levels of doping can adversely affect the efficiency and overall performance of the solar cells. Furthermore, the analysis reveals that solar cells incorporating a MoS_2_ buffer layer are more sensitive to increased acceptor concentration compared to those utilizing a WS_2_ buffer layer. These findings align with previous research documented in the scientific literature^37^, highlighting the importance of buffer layer material in optimizing the performance of doped solar cells. The carrier concentration in the Cu_2_SrSnS_4_ absorber layer is influenced by acceptor impurities, as well as any deviations from stoichiometry or defects in the synthesized material^38^.

**Fig. 7:**
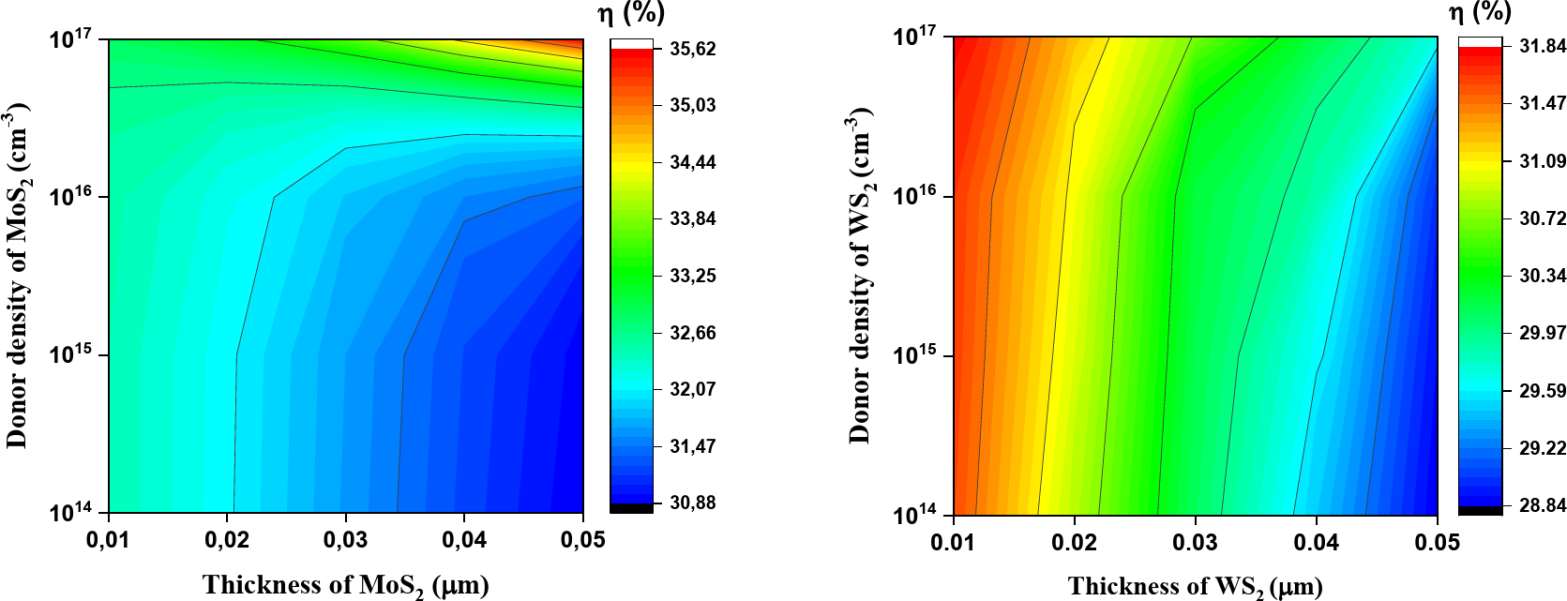
Contour plots of efficiency dependence of thickness and donor density of buffer layer.

The variation of the solar cell efficiency as a function of thickness and donor density of the buffer layer is shown as two-dimensional (2D) contour plots in Fig. 7. Both the performance of Cu_2_SrSnS_4_-based solar cells and their dependence on the thickness and donor density of MoS_2_ and WS_2_ are evident. We conclude that the conversion efficiency of the solar cell with the MoS_2_ buffer layer is higher than that with the WS_2_ buffer layer. Additionally, photovoltaic efficiency remains low at low donor densities, regardless of the buffer layer thickness. This is attributed to the reduced contribution of photo-generated carriers in the n-type quasi-neutral regions, as most electron-hole pairs recombine in this zone before they can reach the depletion region^39^.

**Fig. 8:**
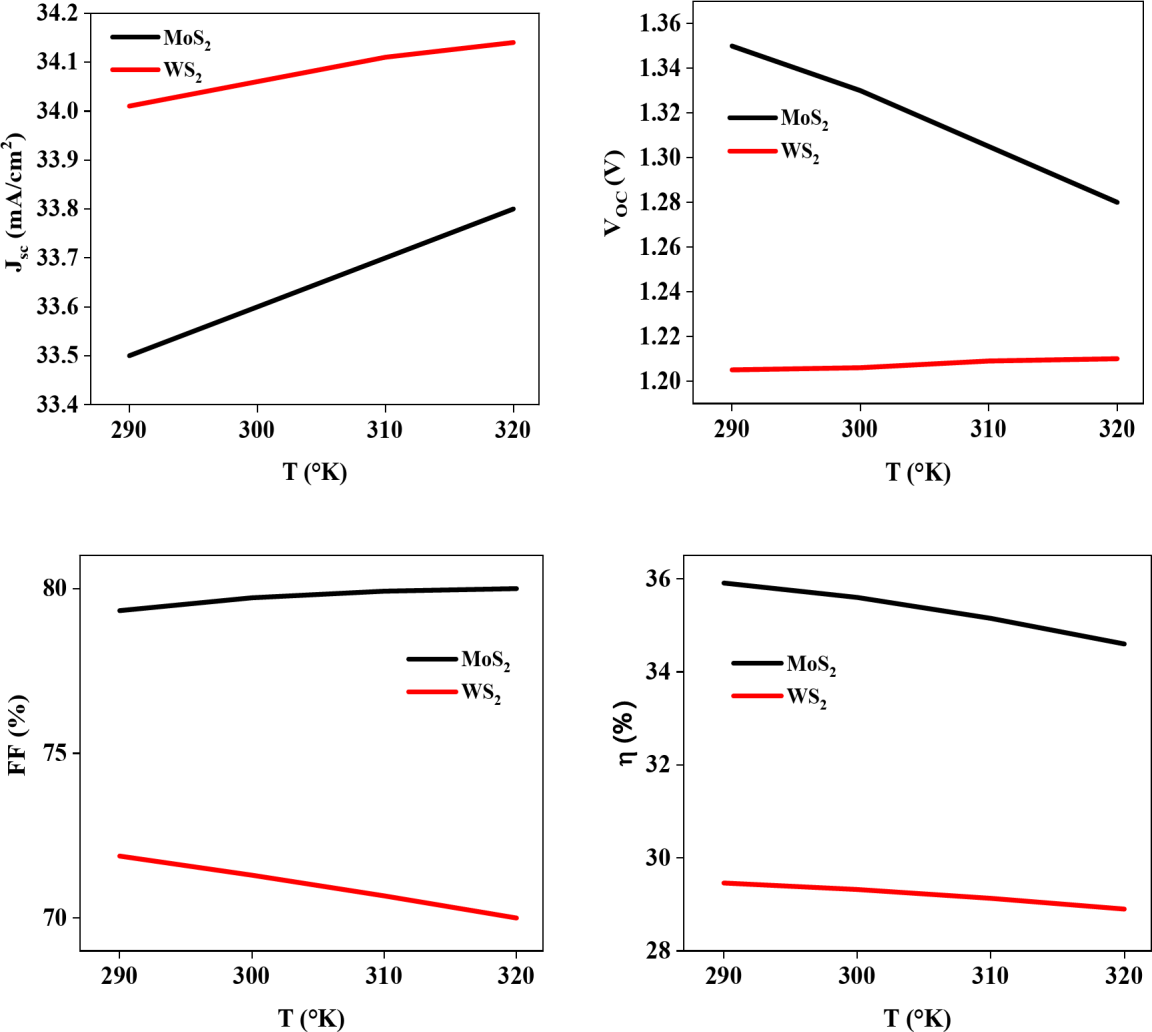
Variation of temperature as a function of the photovoltaic parameters.

The effect of temperature on Cu_2_SrSnS_4_ based solar cells with different buffer layers was analyzed by changing the temperature from 290 K to 320 K. The results of this investigation are illustrated in Fig. 8.

As temperature increases, the reduction in bond energy, driven by the higher velocity of charge carriers, leads to a narrowing of the semiconductor band gap. This change results in a higher recombination rate of electrons and holes, causing a drop in open-circuit voltage (V_OC_)^40^. The reverse saturation current also rises with temperature, further reducing both V_OC_ and the fill factor (FF)^41^. The combined decrease in short-circuit current density (J_SC_), fill factor (FF), and open-circuit voltage (V_OC_) ultimately lowers the overall efficiency. Consequently, the efficiency of Cu_2_SrSnS_4_ based solar cells with MoS_2_ and WS_2_ buffer layer decreases as the temperature rises.

**Fig. 9:**
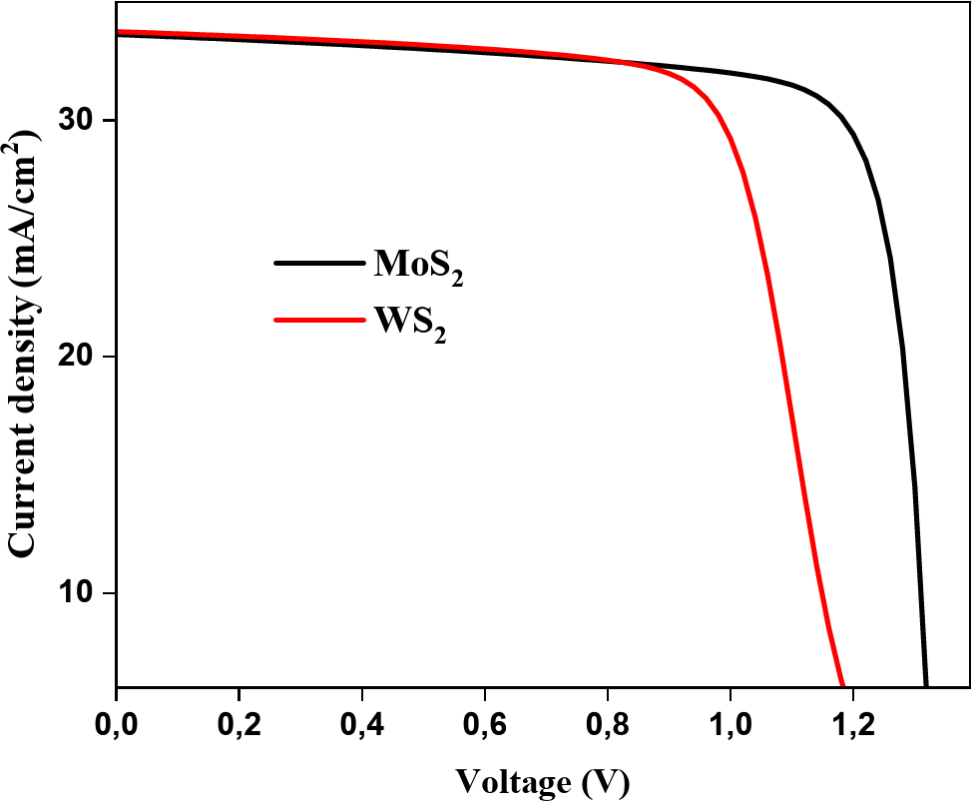
J-V curve of the designed structure.

Figure 9 presents the simulated J-V curve of the designed solar cell with the configuration ZnO/(MoS_2_ or WS_2_)/Cu_2_SrSnS_4_. The simulation was conducted using an energy band gap of 1.79 eV, an acceptor density of 10^16^ cm^− 3^ for Cu_2_SrSnS_4_, and Cu_2_SrSnS_4_ layer thickness of 3 μm, all at an operating temperature of 300 K. The results demonstrate that the ZnO/MoS_2_/Cu_2_SrSnS_4_ and ZnO/WS_2_/Cu_2_SrSnS_4_ devices achieved impressive power conversion efficiencies of 35.6% and 29.1%, respectively. These efficiency levels highlight the potential of these configurations for high-performance solar cell applications, making them promising candidates for future research and development in renewable energy technologies^42^.

MoS_2_ outperforms WS_2_ as a buffer layer in optoelectronic devices due to its bandgap, higher carrier mobility, and smoother, less defective films. These enhance charge transfer, reduce recombination losses, and improve interface quality. MoS_2_’s superior mechanical and thermal stability further solidifies its preference for photovoltaic applications^43,44^.

Table 2 presents a comprehensive summary of the optimal results attained in this investigation, alongside key performance parameters of solar cells from numerical and experimental studies available in the literature for comparison. This table shows that the results we obtained were comparable to the literature’s experimental values. By comparing parameters such as short-circuit current density (J_SC_), open-circuit voltage (V_OC_), and overall efficiency (η), it becomes evident that the performance metrics from our study are comparable to or even exceed certain experimental benchmarks. This comparison reinforces the efficacy of SCAPS-1D as a powerful tool for forecasting solar cell performance under realistic conditions, making it valuable for both predictive modeling and the design of next-generation photovoltaic devices. Furthermore, these results provide valuable insights into optimizing material properties and device architectures, facilitating improvements in solar cell technologies^45^.

**Table 2:** Comparative analysis of the proposed Cu_2_SrSnS_4_ based solar cells against previously reported cells.

Configuration	J_sc_(mA/cm^2^)	V_oc_ (V)	η (%)	Ref.
ITO/ZnO/CdS/CSTS/Mo	3.80	0.38	0.59	^46^
Ag/ITO/ZnO/CdS/CSTS/Mo	1.63	0.29	0.16	^15^
graphene/MoS_2_/CZTS/Ni	25.3	0.8521	18.27	^32^
i-Zno/WS_2_/CIGS/Mo	29.57	1.026	23.4	^47^
AZO/ZnO/ZnS/CSTS/Mo	~ 39	0.62	20.12	^24^
ZnO/MoS_2_/CSTS/Mo	33.62	1.32	35.6	This work
ZnO/WS_2_/CSTS/Mo	33.75	1.18	29.1	This work

**Fig. 10:**
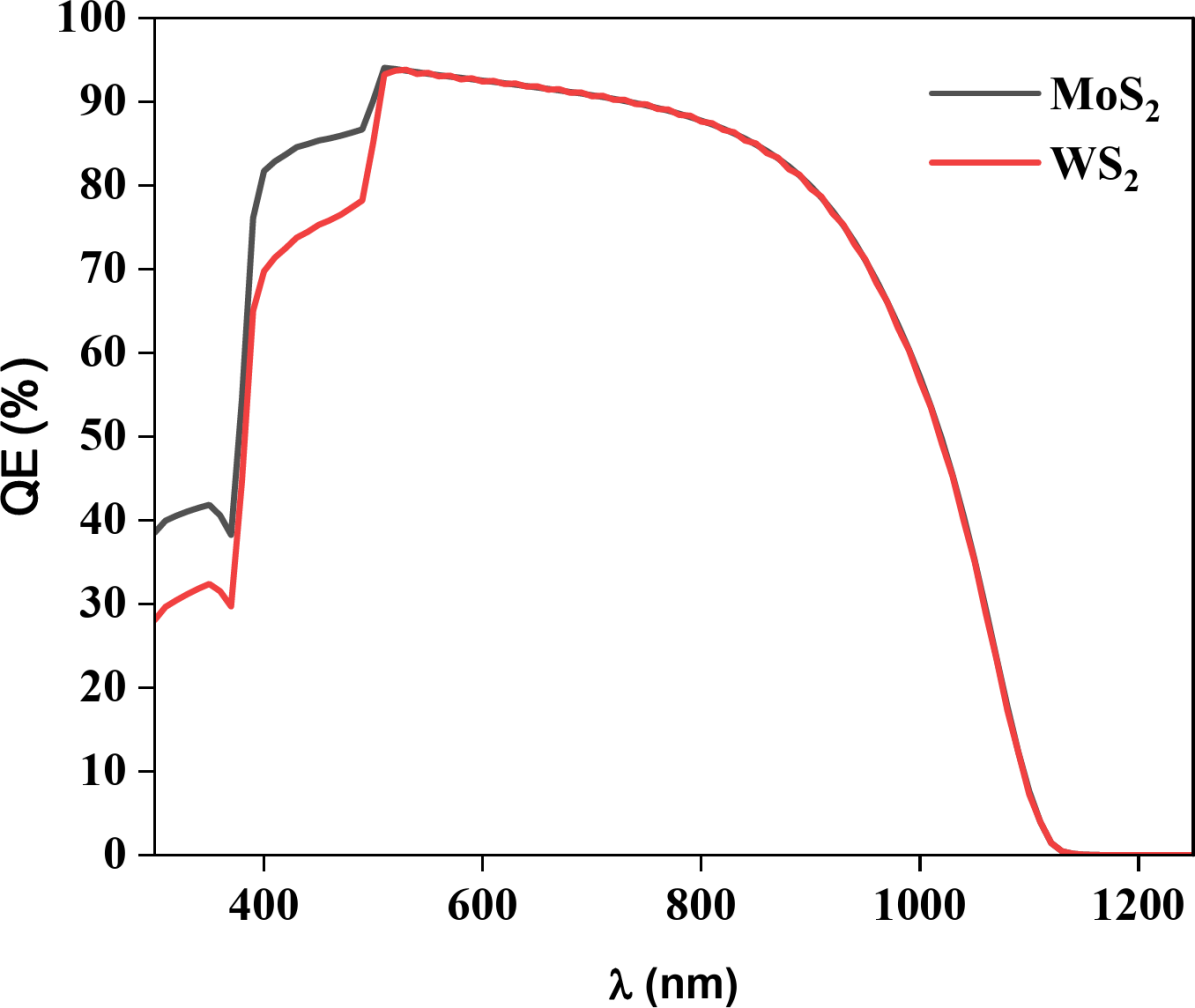
Quantum Efficiency of ZnO/(MoS_2_ or WS_2_)/Cu_2_SrSnS_4_ solar cells.

The graph in Fig. 10 illustrates the variation in quantum efficiency for MoS_2_ and WS_2_ buffer layers-based structures over a range of wavelengths from 300 nm to 1250 nm. MoS_2_ exhibits a higher quantum efficiency than WS_2_ across most of the wavelength spectrum, particularly in the shorter wavelength range. Both materials show an increasing quantum efficiency with longer wavelengths, with MoS_2_ maintaining superior performance throughout^48^. This data highlights the potential of MoS_2_ as a more efficient buffer layer compared to WS_2_ in photovoltaic applications.

## Conclusions

This study highlights the significant potential of Cu_2_SrSnS_4_ (CSTS) as an effective absorber material for high-efficiency solar cells, especially when combined with transition metal dichalcogenide (TMD) buffer layers like MoS_2_ and WS_2_. SCAPS-1D simulations demonstrate that the MoS_2_ buffer layer surpasses WS_2_, attaining a remarkable power conversion efficiency (PCE) of 35.6%, in contrast to 29.1% for WS_2_-based devices. The improved performance of MoS_2_ is due to the adjustment of numerous critical factors, such as absorber layer thickness and doping concentration, which greatly affect efficiency. The study indicates that augmenting the thickness of the CSTS absorber layer enhances light absorption and overall device efficiency, however high doping concentrations result in efficiency decline due to increased recombination rates. Additionally, the research of temperature dependence reveals a decline in performance at higher temperatures, underscoring the necessity of integrating temperature-stabilizing mechanisms into forthcoming device designs. The results indicate that CSTS, due to its advantageous defect tolerance and stability, serves as a feasible alternative to traditional materials like CZTS, addressing issues such as open-circuit voltage constraints. The integration of CSTS with MoS_2_ as a buffer layer presents a viable approach for the development of high-efficiency, stable solar cells, with significant implications for the advancement of photovoltaic technology.

## Data Availability

The datasets used and/or analyzed during the current study are available from the corresponding author upon reasonable request.
